# Phase Transition Process of Graphite to Diamond Induced by Monodispersed Tantalum Atoms at Ordinary Pressure

**DOI:** 10.1002/advs.202411504

**Published:** 2025-01-22

**Authors:** Zhiguang Zhu, Chengke Chen, Shaohua Lu, Xiao Li, Xiaojun Hu

**Affiliations:** ^1^ College of Materials Science and Engineering Zhejiang University of Technology Hangzhou 310014 P. R. China; ^2^ Moganshan Diamond Research Center De Qing Huzhou 313200 P. R. China; ^3^ Diamond Joint Research Center for Zhejiang University of Technology and Tanghe Scientific & Technology Company De Qing Huzhou 313200 P. R. China; ^4^ Moganshan Institute ZJUT De Qing Huzhou 313200 P. R. China

**Keywords:** annealing, diamond, graphite, ordinary‐pressure, phase transition

## Abstract

The transformation of graphite into diamond (2–10 nm) at ordinary pressure by monodispersed Ta atoms was recently reported, while the effects of Ta concentration on the transition process remain obscure. Here, by regulating the Ta wire treatment time, as well as the annealing time and temperature, larger diamond grians (5–20 nm) are successfully synthesized, and the transition process of graphite to diamond is revealed to vary with Ta concentration. Specifically, short Ta wire treatments (5–10 min) induce graphite to form a “circle” structure and transforms into diamond directly after annealing. Long Ta wire treatments (15–25 min) produce larger and more “circle” structures, containing an increased number of graphite layers. After annealing at 1100 °C for 30–120 min, graphite first transforms into amorphous carbon, then to i‐Carbon and n‐Diamond, and finally to diamond. Notably, a large amount of n‐Diamond and diamond are formed after 120 min annealing. By modulating the annealing temperature from 500 to 1200 °C for 30 min, diamond is already obtained at 500 °C, and hexagonal diamond up to 20 nm in size at 1200 °C. This provides a fresh insight into the graphite/diamond transition process and an approach for diamond synthesis.

## Graphical abstract

Graphite transforms to diamond at ordinary pressures by monodisperse Ta atoms, with the process varying with Ta concentration. The short Ta‐wire treatment induces graphite to form a circle structure, which transforms directly into a diamond. Long Ta‐wire treatment lets graphite curl to larger circle structures, which transform to amorphous carbon, then to i‐Carbon and n‐Diamond, and finally to diamond.

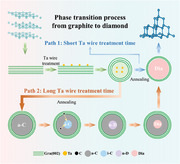

## 1 Introduction

Carbon has three hybridization modes (sp, sp^2^, and sp^3^) and forms ≈500 allotropes, ranging from 0 to 3D structures,^[^ ^1^ ^]^ including C_60_,^[^ ^2^, ^3^ ^]^ graphene,^[^ ^4^, ^5^, ^6^ ^]^ graphite,^[^ ^7^, ^8^ ^]^ carbon nanotubes,^[^ ^9^, ^10^ ^]^ and diamond.^[^ ^11^, ^12^ ^]^ Among them, diamond is the material with the greatest hardness, the best thermal conductivity, and the widest light transmission band, which plays an important role in the fields of industrial processing, electronic information, and public health.^[^ ^13^, ^14^ ^]^ In addition, diamond has many excellent properties such as wide bandwidth, high carrier mobility, and high breakdown voltage, which is known as the “ultimate semiconductor” and is expected to achieve high‐power, high‐frequency, and low‐loss electronic device preparation.^[^ ^15^, ^16^, ^17^, ^18^ ^]^

Natural diamonds are difficult to mine and expensive, while graphite is plentiful and cheap. So, various methods have been developed to promote the phase transition from graphite to diamond, achieving the transition of sp^2^ to sp^3^ carbon. At present, the commonly used industrial technology for achieving this transition is the high pressure and high temperature (HPHT) method, which simulates the natural process of diamond formation underground by providing pressure and temperature conditions over 10 GPa and 3000 °C, respectively, allowing the carbon atoms in graphite to recombine into the structure of diamond.^[^ ^13^, ^19^, ^20^, ^21^, ^22^ ^]^ The extreme conditions make high demands on the equipment and lead to high preparation costs. Conversely, the chemical vapor deposition (CVD) technique allows the epitaxial deposition of diamond in the metastable P‐T region with very low‐pressure conditions (1–800 torr) by cracking the gaseous carbon source and hydrogen into hydrocarbon groups and atomic hydrogen at high temperature (500–800 °C).^[^ ^23^ ^]^ The most common methods are hot filament CVD (HFCVD) and microwave plasma CVD (MPCVD).^[^ ^24^, ^25^, ^26^, ^27^ ^]^ However, these methods still require stringent conditions and high costs to grow diamonds.

Our previous research found that nanographite and amorphous carbon at the grain boundaries of nanocrystalline diamond (NCD) films grown by HFCVD transformed to the diamond after annealing at ordinary pressure, and demonstrated the key role of monodispersed Ta atoms in the phase transition process using first‐principles calculations.^[^ ^28^ ^]^ Furthermore, we discovered the transformation of vertical graphene sheets to diamond on the diamond surface in HFCVD and proposed the mechanism that diamond was formed by the phase transition of graphite in the H, O, and Ta complexing atmosphere, overturning the traditional view that diamond was formed by the accumulation of carbon‐containing groups in CVD.^[^ ^29^ ^]^ Based on these, we deposited Ta atoms directly on the surface of graphite flakes and used the action of monodispersed Ta atoms to induce the transformation of graphite into diamond at low pressures.^[^ ^30^ ^]^ It was found that annealing prompts the diffusion of monodispersed Ta atoms and forms a coexistence region of amorphous carbon (a‐C), i‐Carbon (i‐C), and diamond within the graphite, developing a new method of preparing diamond based on graphite at ordinary pressure.^[^ ^30^ ^]^ However, the high power and long treatment time for depositing Ta atoms in this process led to the formation of massive TaC phases, which decreases the number of monodispersed Ta atoms loaded on the graphite, and only yields a small amount of diamond with 2–10 nm. Also, although amorphous carbon, i‐C, and diamond were detected in graphite, the phase transition process, i.e., the relationship of these phases remains unclear. Among the parameters that affect the phase transition process, the concentration of Ta deposited on the graphite is the key factor, while its effects on the phase transition process are indistinct.

In this work, we have reduced the TaC content and synthesized more and larger (5–20 nm) diamonds by decreasing the Ta wire treatment power as well as time to vary the concentration of Ta deposited on graphite, and investigated the effects of Ta concentration on the graphite phase transformation. Then, we adjusted the annealing time and temperature to further clarify the phase transition process. It is found that for shorter Ta wire treatment time (5–10 min), monodisperse Ta atoms cause the graphite to curl and form a “circle” structure, and the internal graphite is directly transformed into a diamond. By extending the Ta wire treatment time from 10 to 20 min, the concentration of Ta increases, and the number and size, as well as graphite layers of the “circle” structure, increase with the treatment time. At this point, graphite is transformed first into amorphous carbon, then into i‐C and n‐Diamond (n‐D), and finally into diamond. By annealing Ta‐loaded graphite flakes at 1100 °C for 30–120 min, it is noted that i‐C, the diamond variant structures, appears at 30 min and n‐D at 120 min. Notably, a large amount of n‐Diamond and diamond are formed after 120 min annealing. Also, increasing the annealing temperature from 500 to 1200 °C, shows that annealing at the lower temperatures of 500 and 700 °C for 30 min already achieves diamond, and hexagonal diamonds are obtained at 1200 °C with diamond sizes up to 20 nm. This develops a different method for preparing diamonds from the conventional HPHT and CVD methods and provides a new strategy for diamond preparation using other different carbon materials as precursors.

## 2 Results and Discussion

### 2.1 Effects of Ta Concentration on Graphite Structure and Phase Transition Process

Figure S1 (Supporting Information) shows the morphology of the intrinsic graphite and the samples with different Ta wire treatment time from 5 to 25 min by field emission scanning electron microscope (FESEM). It is observed that the surface of the intrinsic graphite flake is relatively flat with only a few holes and cracks. After the Ta wire treatment for different time, the surfaces are slightly rougher, while in general there are no particularly obvious changes. High‐resolution transmission electron microscope (HRTEM) graphs in Figure S2 (Supporting Information) show that the intrinsic graphite displays a smooth layered structure, containing straight banded stripes of graphite (002) crystal planes and a large distribution of (100) crystal planes. The inset selected area electron diffraction (SAED) image in SAED‐S2a (Supporting Information) displays graphite (002), (100), (110), and (112) planes.

**Figure 1:**
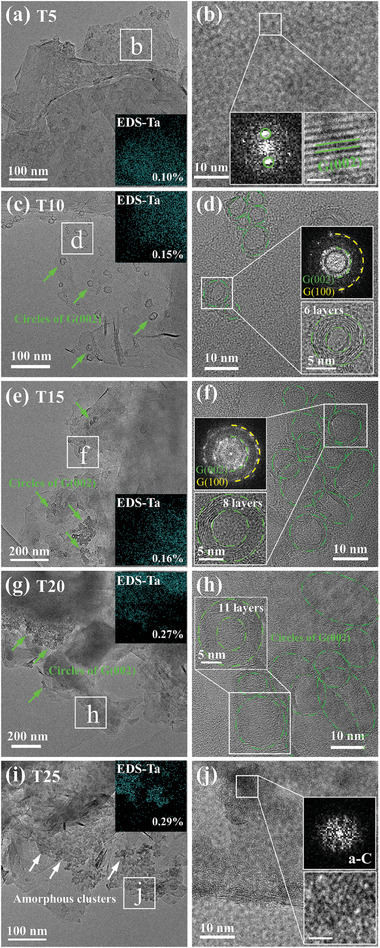
HRTEM images of samples with different Ta wire treatment time: a,b) 5 min; c,d) 10 min; e,f) 15 min; g,h) 20 min; i,j) 25 min. The white line in the inset figure indicates the 1 nm scale.

HRTEM images in **Figure** **1** show the structures of the samples treated by Ta wire from 5 to 25 min. It is found that for the Ta wire treated sample by 5 min (Sample T5), its structure is similar to intrinsic graphite (Figure 1a), with striped lattice stripes of graphite (002) plane appearing at the edges (Figure 1b). The diffraction information of image SAED‐a is also similar to the intrinsic graphite, as shown in Figure S3a (Supporting Information). The inset Energy‐dispersive X‐ray spectroscopy (EDS) spectrum EDS‐Ta indicates the dispersed presence of Ta in the region with ≈0.10 at%. It demonstrates that the Ta atoms escaping from the tantalum wire are successfully deposited on the graphite substrate.

With increasing treatment time to 10 min (sample T10), the diffraction rings displayed in Figure S3b (Supporting Information) correspond to graphite (002), (100), (101), and (110) crystal planes, indicating that the region is still graphite. Especially, there are some independently existing “circle” structures (green arrows) emerging in the thin region of sample T10 (Figure 1c). By enlarging the d area in Figure 1c, as shown in Figure 1d, it is observed that the “circle” structures are formed by the bending of the graphite (002) lattice with the crystal plane spacing of 0.334 nm and that each “circle” contains ≈6 layers of graphite (002) planes. The inset Fourier Transform (FT) image displays the diffraction information of the graphite (002) and (101) crystal planes. Notably, dispersed Ta atoms are also detected in this region with the contents of 0.15 at%. This suggests that the new “circle” structure formed by the curling of graphite (002) is related to Ta atoms deposited on the graphite.

Similar structures are observed with treatment time increasing to 15 and 20 min (samples T15 and T20), as shown in Figure 1e–h. The “circle” structures in these samples become denser and the crystal planes increase to 8–11 layers (Figure 1f and 1h). This suggests that the number and size of the “circle” structures, and the number of layers of graphite (002) contained in the structures, increase with the processing time. The inset maps EDS‐Ta demonstrate that the monodispersed Ta content increases to 0.16 at% and 0.27 at% in the samples T15 and T20, respectively. This indicates that the concentration of Ta in graphite increases with extending processing time, increasing the graphite layers in the “circle” structures.

When the Ta wire treatment time is further increased to 25 min (sample T25), many clusters are present in the thin region, as seen in Figure 1i. The absence of ordered lattice stripes in the clusters and the corresponding FT image shows dispersed diffraction rings (Figure 1j), proving that these clusters are amorphous carbon. Also, Figure S3e (Supporting Information) only shows the diffraction rings of the graphite crystal planes. The inset EDS‐Ta in Figure 1i indicates that the element Ta is no longer dispersed but concentrated in the cluster regions and the content increases to 0.29%. That is, the increase in the number of Ta atoms and their aggregation leads to the formation of amorphous carbon clusters.

The above results show that the morphology and structure of the graphite substrate change differently after various Ta wire treatment time, which is related to the state and concentration of Ta atoms. Specifically, after 5 min treatment, sample T5 remains similar to the intrinsic graphite due to the short treatment time and limited concentration of Ta. With treatment time increasing to 10–20 min, samples T10, T15, and T20 all have the “circle” structures formed by the curling of graphite (002), the number and size of which increased with treatment time. As the tantalum wire treatment time is further increased to 25 min, the higher concentration of Ta led to the production of many amorphous carbon clusters. That is, with extended treatment time, the increase of monodisperse Ta atoms promotes the curling of graphite into a “circle” structure, whose size and number of graphite layers increase, but longer treatment time lead to aggregation of Ta atoms and the formation of amorphous carbon clusters. It is also noted that there is no TaC found in these samples with different Ta wire treatment time, indicating that reducing the power and time of Ta wire treatment effectively reduces the formation of TaC. However, no diamond is found in the samples, suggesting that no phase transition from graphite to diamond has occurred after Ta wire treatment.

**Figure 2:**
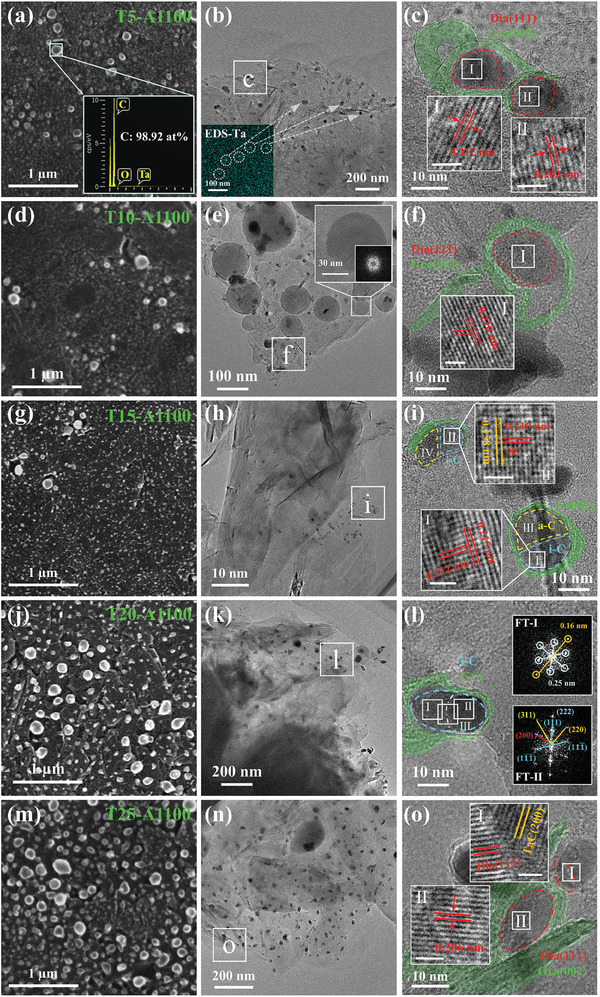
FESEM and HRTEM images of samples with different Ta wire treatment time after annealing at 1100 °C for 30 min: a–c) 5 min; d–f) 10 min; g–i) 15 min; j–l) 20 min; m–o) 25 min. The white line in the inset figure indicates the 1 nm scale.

In order to achieve the transformation of graphite to diamond, we have performed vacuum annealing on the graphite and Ta wire‐treated samples at 1100 °C for 30 min. The surface morphology of these samples is shown in **Figure** **2**. It is obvious that spherical nanoparticles are formed on the surface of samples treated with tantalum wire at different time after annealing. The FESEM‐EDS spectrum of the nanoparticles (inset in Figure 2a) shows that the main component is element C with an atomic percentage of 98.92 at%, suggesting that these nanoparticles are carbon spherical particles. Meanwhile, the size of the dense carbon spherical nanoparticles first decreases (samples T5‐T15) and then increases (samples T15‐T25) with the increase of Ta wire treatment time.

HRTEM tests were performed on these annealed samples to further analyze their structure and phase composition. Figure 2b shows the formation of some black nanoparticles after annealing of the Ta wire treated 5 min sample (T5) under 1100 °C for 30 min (sample T5‐A1100). It is observed that diffraction information of the graphite (002), (100), (110), and TaC (111), (220) crystal planes are present in Figure S4a (Supporting Information). Meanwhile, the EDS‐Ta confirms the presence of Ta atoms in the sample and its enriched zones in some black nanoparticles. These indicate that some of the black particles are TaC. Further magnification of the black nanoparticles of region c in Figure 2b reveals distorted graphite (002) lattice stripes (green regions). It is similar to the “circle” structure found in the unannealed samples T10, T15, and T20, but the number of curled graphite layers increases significantly after annealing. Concurrently, black nanoparticles are formed within the graphite “circle” structure, and the lattice stripes within the particle have a crystal plane spacing of 0.212 nm, close to the graphite (100) and the diamond (111) planes. Based on the TEM analyses of the intrinsic graphite, Ta wire treated, and annealed samples in Figure S5 (Supporting Information), the inverse Fourier transform (IFT) of the diffraction points from the graphite (100) crystal planes indicates that the graphite (100) planes are generally distributed as large areas in the sample rather than appearing as isolated particles. Figure S5c (Supporting Information) shows that the lattice orientation inside the particle is significantly different from that of the surrounding graphite and exists only inside the particle. In addition, TaC has no planes with spacing close to 0.212 nm, and the diffraction intensity of the planes close to this spacing for Ta and other carbides is extremely weak. Therefore, the 0.212 nm lattice stripe belongs to the diamond (111) crystal planes, and particle I is a diamond particle ≈12 nm in size. Similarly, region II exhibits the lattice stripes with a spacing of 0.202 nm, close to the diamond (111) planes, suggesting that it is also a diamond particle, ≈10 nm in size. These results demonstrate the formation of diamond nanoparticles ranging from 10–12 nm in the sample, which are larger than those formed in the sample from the high‐power and long‐time Ta wire treatment.^[^ ^30^ ^]^ It is also illustrated that monodispersed Ta atoms are distributed on the graphite surface, causing the graphite to transform directly into diamond after annealing. This is consistent with our previous theoretical calculations, which found that graphite was directly transformed to diamond spontaneously in the presence of monodisperse Ta atoms.^[^ ^28^ ^]^

Figure 2e shows the HRTEM image of the sample T10 after annealing at 1100 °C for 30 min (T10‐A1100), where similar black nanoparticles and more spherical particles larger than 100 nm in size are formed. This is generally consistent with the spherical nanoparticles observed in SEM results. The inset FT image in Figure 2e suggests that the carbon spherical particles have amorphous structure characteristics. The diffraction rings in Figure S4b (Supporting Information) correspond to the information of graphite (002), (100), (110), (112) and TaC (111), (200), (220), (311) planes, suggesting that TaC is formed after annealing. Enlarging the region f in Figure 2e, as shown in Figure 2f, we find that nanoparticles surrounded by graphite (002) are also present in sample T10‐A1100, with a spacing of 0.210 nm inside the particle, belonging to the diamond (111) planes, revealing diamond particles ≈15 nm in size exist in the sample. These results show that the graphite inside the “circle” transits directly to the diamond after annealing.

Sample T15‐A1100 also forms black nanoparticles in the graphite region (Figures 2h and S4c (Supporting Information)) indicates that this region is still mainly graphite. Enlarging the region i in Figure 2h, as shown in Figure 2i, we find that there exist similar two particles which are both encapsulated by graphite (002) crystal planes. Both particles are internally divided into two regions, one of which has clear lattice stripes, such as regions I and II. Specifically, the spacing of the lattices is 0.212 and 0.215 nm in region I as well as 0.210 and 0.158 nm in region II, respectively. Meanwhile, the angles θ_1_ (0.212/0.215) and θ_2_ (0.210/0.158) are both 90°, corresponding to i‐C (200)/(020) and (200)/(022) planes, respectively. The i‐C structure was formed by removing some of the internal C atoms from the cubic diamond lattice and had a body‐centered structure with space group P2_1_3 or P4_2_32 and lattice parameters of 0.425–0.432 nm.^[^ ^31^, ^32^, ^33^, ^34^ ^]^ Meanwhile, H atoms may have entered the interior of its crystal structure and enhanced its stability.^[^ ^35^, ^36^, ^37^ ^]^ This structure is defective compared to the perfect cubic diamond structure, which is considered a transition state from graphite to diamond. In contrast, other regions (III and IV) in the particles have no ordered lattice and exhibit the characteristics of amorphous carbon, as shown in Figure S6 (Supporting Information). That is, these lattice‐incomplete particles are probably in the process of transformation, suggesting that amorphous carbon and i‐C are intermediate structures in the phase transition. However, there is no direct transition of graphite to diamond found in this sample.

The low magnification HRTEM image of sample T20‐A1100 is similar to that of the previous sample, as shown in Figure 2k. The SAED information in Figure S4d (Supporting Information) suggests that the sample T20‐A1100 is dominated by graphite and TaC. By enlarging the nanoparticle in region l of Figure 2k, it has lattices of about 0.250 and 0.160 nm internally and is surrounded by the graphite (002) lattice externally (Figure 2l). However, the crystal angles are different in regions I and II, as shown in images FT‐I and FT‐II. The lattices of region II correspond to i‐C, but region I is not consistent with either graphite, diamond, or i‐C. Then, enlarging the junction region III, as shown in Figure S7 (Supporting Information), we find that the lattice orientation is shifted, proving that the lattices are different in the two regions and that the nanoparticle is in the transition process. Compared to the particles of Figure 2i, the amorphous carbon transforms to form an ordered lattice and gradually satisfies the i‐C structure. Similarly, the direct transition of graphite to diamond is absent.

For the sample T25‐A1100, the number of black nanoparticles increases in the thin region of graphite (Figure 2n), and the diffraction rings of graphite and TaC appear in Figure S4e (Supporting Information). In Figure 2o, there are two lattice planes inside the particle I with the spacing of 0.227 and 0.212 nm, which are close to the TaC (200) and diamond (111) planes, respectively. On the contrary, only lattice planes with a spacing of 0.206 nm are present inside particle II, which is consistent with the crystal plane spacing of the cubic diamond (111) planes. Moreover, there are lattice streaks with a spacing of 0.334 nm on the edges of the particle, which suggests that particle II is a diamond particle surrounded by the graphite (002) planes. Thus, diamonds with 5–15 nm surrounded by graphite (002) and coexisting with TaC are found in sample T25‐A1100.

The above results indicate that graphite flakes with different Ta wire treatment time partially transform into 5–15 nm diamond after annealing at ordinary pressure. For the short time of Ta wire treatment (5–10 min), the concentration of Ta is low, and the monodispersed Ta atoms on the graphite surface cause the graphite to curl and form a separate “circle” structure, and the internal graphite is directly transformed into diamond after annealing. By extending the Ta wire treatment time (15–20 min), the concentration of Ta increases, and the increased monodispersed Ta atoms on the graphite surface transform the graphite inside the densely packed “circle” structure into amorphous carbon first, and then a diamond with defects is formed. Notably, the diamond and i‐C particles in the annealed samples have amorphous carbon near them and are surrounded externally by graphite (002) crystal planes, which is similar to the “circle” structure in the unannealed samples. Moreover, the large concentration of Ta in the long‐time Ta wire treatment samples led to the direct transition of graphite to amorphous carbon and further transition to diamond in the subsequent annealing. So, we believe that when loaded with appropriate concentrations of Ta, monodisperse Ta atoms transform graphite directly into diamond; and with larger concentrations of Ta, monodisperse Ta atoms cause graphite to curl, amorphous carbon to form and transform into a diamond defect structure, resulting in the formation of i‐C particles surrounded by graphite (002) planes.

### 2.2 Effects of Different Annealing Time on the Graphite/Diamond Phase Transition

The various phase transition paths and products of graphite loaded with different concentrations of Ta occur during annealing, indicating that annealing plays an important role in achieving the transformation from graphite to diamond. Meanwhile, it is unclear what the process of further transition from amorphous carbon and i‐C to diamond. Therefore, we selected the Ta wire‐treated 15 min sample to further investigate the effects of different annealing time on the phase transformation. Figure S8 (Supporting Information) shows the surface morphology of the samples annealed at different time, all of which form nanospherical carbon particles on the surface. With increasing annealing treatment time, the number of particles form on the surface decreases, but the size gradually increases. This indicates that the annealing time affects the number and size of nanoparticles formed on the surface of the samples.

**Figure 3:**
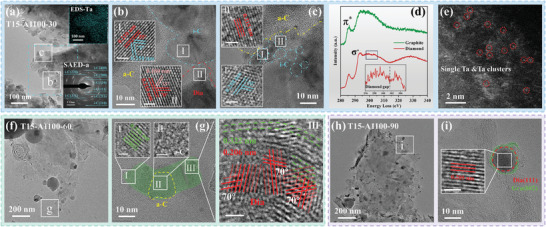
HRTEM images of samples with different annealing time: a–c) 30 min, f,g) 60 min, h,i) 90 min; the insets are corresponding SAED images, EDS spectrum of Ta element, and magnified images corresponding to the square regions; (d) EELS spectra and (e) HAADF images of sample T15‐A1100‐30. The white line in the inset figure indicates the 1 nm scale.

**Figure** **3** shows the HRTEM images of the samples with different annealing time. For sample annealed at 1100 °C for 30 min (T15‐A1100‐30), we observe a special scale‐like region in Figure 3a. Image SAED‐a shows diffraction rings with spacings of 0.300, 0.250, 0.210, 0.180, 0.160, 0.130, and 0.110 nm, corresponding to the i‐C (110), (111), (200) (211), (220), (331) and (400) planes, respectively. In the constituent electron diffraction mode, i‐Carbon also contains reflections forbidden by cubic diamonds, such as the (110), (200), and (211) crystal planes. The information we obtained is close to previously reported data, as shown in Table S1 (Supporting Information). From the high magnification HRTEM images in Figure S9 (Supporting Information), it is found that the sample presents a large area of amorphous carbon, indicating that more graphite is transformed into amorphous carbon. In addition, region I in Figure 3b shows lattice stripes with spacings of 0.250 and 0.210 nm, respectively, with corresponding crystal angles θ_1_ (0.250/0.210) of 55° and θ_2_ (0.250/0.250) of 70°, which correspond to the i‐C (111), (11$\bar{1}$) and (200) planes. The other region II contains the lattice stripes with spacings of 0.206 and 0.180 nm, with crystal angles θ_1_ (0.206/0.206) of 70° and θ_2_ (0.206/0.180) of 55°, corresponding to diamond (111), (11$\bar{1}$), and (200) planes. Also, other special lattices are formed in amorphous carbon, as shown in Figure 3c. For example, particle I have internal lattice stripes with a spacing of 0.250 nm and a crystal angle of 70°, which satisfies the i‐C (111) planes. Particle II contains i‐C (200) planes with a spacing of 0.215 nm. These indicate that the scale‐like region contains a large amount of amorphous carbon in which many i‐C and diamonds are formed. Considering the positive effect of Ta atoms on the phase transition, the distribution of Ta elements within the blue line area in Figure 3a is detected, as shown in the inset EDS spectrum. It is evident that Ta atoms in the fish scale‐like region remain monodispersed, which is the reason for the larger transition in this region. This confirms that monodispersed Ta atoms are more favorable for the phase transition, and amorphous carbon and i‐C are the transition states from the graphite phase to diamond.

Further, the electron energy loss spectroscopy (EELS) spectrum of sample T15‐A1100‐30 is obtained by spherical aberration‐corrected transmission electron microscope (Figure 3d). There are two typical EELS results for this sample, one spectrum (green) originated from an untransformed graphite substrate with a strong π^*^ peak at 285.5 eV associated with graphite. The other spectrum (red) shows a strong σ^*^ peak at 293.2 eV and a tiny valley at 301.6 eV, indicating the presence of a small amount of diamond in the sample. At the same time, the high‐angle annular dark field (HAADF) image shows that many monodisperse Ta atoms and clusters of Ta atoms are present in the sample after the Ta wire treatment (Figure 3e). This demonstrates that monodisperse Ta atoms are efficiently deposited on graphite by the HFCVD system, which plays an important role in the graphite transition process.

In the HRTEM image of the sample T15‐A1100‐60, the familiar black nanoparticles and amorphous carbon sphere particles are present in the thin region (Figure 3f). The diffraction rings of graphite (002), (100), and (110) planes, and diffraction points of TaC (111), (220), and (311) planes are displayed in Figure S10a (Supporting Information). Enlarged region g in Figure 3f is shown in Figure 3g, which displays a curved graphite (002) lattice band (region I). Interestingly, the amorphous carbon region (region II) and lattice transition region (region III) are present at the location of the graphite (002) lattice band bending. It is worth mentioning that there are complex and chaotic lattices with spacings of 0.322 and 0.206 nm in region III. Among them, the planes with a spacing of 0.322 nm are smaller than that of graphite (002) planes, suggesting that the graphite is in the process of distorting. In addition, there are several lattices with a spacing of 0.206 nm and a lattice angle of 70°, corresponding to the cubic diamond (111), just below the distorted graphite. It indicates that carbon atoms in different layers of graphite combine with each other and transform into diamond (111) planes. Thus, it is found that graphite bends to form amorphous carbon and transforms to diamond in the sample T15‐A1100‐60.

When the annealing treatment is extended to 90 min, the number of black nanoparticles increases in the sample T15‐A1100‐90 (Figure 3h). The SAED model in Figure S10b (Supporting Information) shows the signals from graphite and TaC. Among these black nanoparticles, some of them have internal lattice stripes with a spacing of 0.205 nm close to the cubic diamond (111) planes, as shown in Figure 3i. In general, these particles are externally surrounded by graphite (002) planes. That is, diamond particles surrounded by graphite (002) crystal planes are formed after annealing for 90 min, with a size of ≈12 nm.

**Figure 4:**
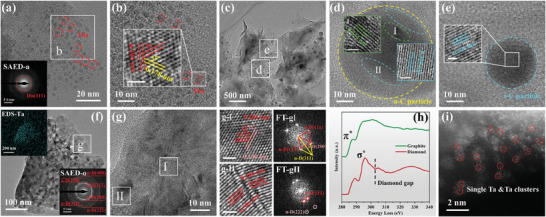
a–g) HRTEM images, the insets are corresponding SAED images, EDS spectrum of Ta element, and magnified images and FT images corresponding to the square regions; h) EELS spectra and i) HAADF images of the sample annealed for 120 min. The white line in the inset figure indicates the 1 nm scale.

**Figure** **4**a‐g shows three different representative regions of the sample annealed at 1100 °C for 120 min (Sample T15‐A1100‐120). First, it is observed that there are many nanoparticles ≈5 nm present in the sample T15‐A1100‐120, as shown in Figure 4a, in which the inset SAED‐a image only displays a lattice diffraction ring corresponding to the spacing of ≈0.206 nm, and other lattice diffractions common to graphite almost disappeared. Enlarged images in Figure 4b give the lattice stripes with the spacing of 0.206 and 0.178 nm in the red circled particles, with θ_1_ (0.206/0.206) of 70°, θ_2_ (0.206/0.178) of 55°, which are consistent with the cubic diamond (111)/(11 $\bar{1}$) and (111)/(200) planes, further confirming the particles are diamond. In addition, all other marked particles also have the diamond (111) lattice. So, the diffraction information in SAED‐a originated from the diamond (111) plane, which indicates that a large amount of graphite transits to the diamond after 120 min annealing.

In the second kind of region in sample T15‐A1100‐120 (Figure 4c), the SAED pattern in Figure S10c (Supporting Information) indicates the presence of graphite and TaC. Magnification of the particle in region d of Figure 4c, as seen in Figure 4d, shows that most part of the particle displays the structure of amorphous carbon with lattice streaks of graphite (002) planes (region I) and i‐C (110) planes (region II) inside the particles. Meanwhile, a complete i‐C particle with a size of ≈30 nm is also found in Figure 4e with the internal lattice stripes of the particle totally corresponding to the i‐C (110) planes. It indicates that amorphous carbon spherical particles have the potential to be completely transformed into i‐C particles. This reveals that amorphous carbon and i‐C are the transition state structures for the transformation of graphite into diamond.

Figure 4f provides the third region of sample T15‐A1100‐120. It is obvious that the SAED image of this region shows the diffraction information of crystal planes with spacings of 0.206, 0.178, 0.125, 0.107, 0.102, and 0.089 nm, which correspond to n‐D (111), (200), (220), (311), (222) and (400) planes, respectively.^[^ ^34^ ^]^ This is consistent with other reports on n‐D, such as Table S1 (Supporting Information). Due to exhibiting reflections such as (200), (222), and (420) which are forbidden in cubic diamond, it is indicated that this is a different structure from cubic diamond. The n‐D is a face‐centered cubic structure formed by removing four carbon atoms inside the cubic diamond structure and possibly including some H atoms inside, with spatial group F $\bar{4}$3m and lattice constant of 3.539–3.596 Å.^[^ ^33^, ^34^, ^35^, ^36^, ^37^, ^38^, ^39^, ^40^ ^]^ Figure 4g is a high‐magnification image of the region g in Figure 4f, where it is clearly shown that the region I has lattice stripes with spacings of 0.206 and 0.180 nm (g‐I). And image FT‐gI contains four pairs of diffraction points with spacings of 0.206, 0.178, and 0.107 nm, and crystal angles θ_1_ (0.206/0.206) of 70°, θ_2_ (0.206/0.178) of 55°, θ_3_ (0.206/0.107) of 30°, and θ_4_ (0.178/0.107) of 25°. It corresponds to the (111), (11$\bar{1}$), (200), and (311) planes of n‐D, respectively. In addition, the magnified image g‐II has two sets of lattice stripes with a spacing of 0.206 nm, and image FT‐gII also shows diffraction points corresponding to the lattice spacing of 0.206 and 0.102 nm, which correspond to n‐D (111) and (222) planes. The inset EDS‐Ta indicates the existence of monodispersed Ta atoms in this region, which plays the key role in achieving a massive phase transition from graphite to diamond.

Furthermore, the EELS spectra of the sample T15‐A1100‐120 are shown in Figure 4h. Obviously, there is a strong graphite‐related π^*^ peak at 285.5 eV, originating from the untransformed graphite substrate. In addition, a strong σ^*^ peak at 293.6 eV and a distinct valley at 301.2 eV are consistent with the diamond features, indicating the presence of diamond in the sample. Compared to annealing for 30 min (sample T15‐A1100, Figure 3d), the intensity of the graphite‐related π^*^ peaks in the EELS spectra decreases and the diamond‐related σ^*^ peaks as well as the valley at 301.2 eV become more significant after extending the annealing time up to 120 min, suggesting more transformation of graphite to diamond. Meanwhile, many monodispersed Ta atoms and clusters of Ta atoms are found in the sample, as shown in Figure 4i, which promoted the transition from graphite to diamond. These results suggest that a large amount of n‐diamond and diamond are formed after 120 min annealing.

Extending the annealing time to 150 min (Sample T15‐A1100‐150), only a large number of TaC particles are observed, with no diamond phase, as shown in Figure S11 (Supporting Information). It clearly suggests that excessive annealing time leads to the production of a large amount of TaC, which reduces the number of monodispersed Ta atoms and limits the occurrence of the phase transition.

### 2.3 Effects of Different Annealing Temperatures on the Graphite/Diamond Phase Transition

The above results suggest that the annealing time has an important effect on the phase transformation. That is, within a certain range of 30–120 min, a longer annealing time is beneficial for the transformation of graphite to diamond. Then, we further adjusted the annealing temperature to study its effects on the phase transformation. Figure S12 (Supporting Information) shows the surface morphology of the samples after annealing at different temperatures in the range of 500–1200 °C for 30 min, with spherical nanoparticles forming on the surface. It is found that the numbers and size of nanoparticles increase with the annealing temperature in the range of 500–1000 °C. However, the size and number of nanoparticles instead decreased after annealing temperature above 1000 °C.

**Figure 5:**
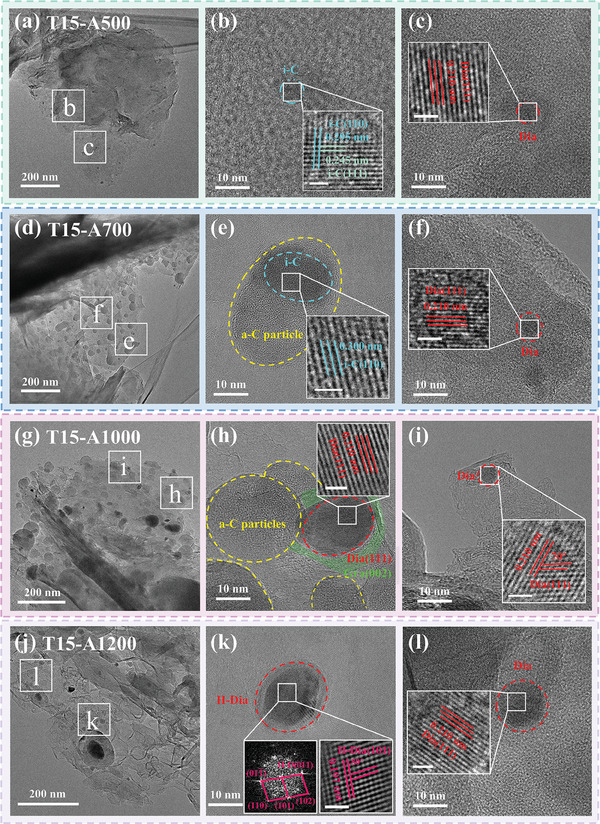
HRTEM images of samples with different annealing temperatures: a–c) 500 °C, d–f) 700 °C, g–i) 1000 °C, and j–l) 1200 °C; the insets are corresponding magnified images and FT images. The white line in the inset figure indicates the 1 nm scale.

The low magnification HRTEM image of the 500 °C annealed sample (T15‐A500) shows that a few black nanoparticles form in the thin region, as seen in **Figure** **5**a. Also, the SAED pattern in Figure S13a (Supporting Information) only shows the diffraction information for the graphite (002), (100), (110), and (112) planes. Figure 5b is the high magnification HRTEM image of region b in Figure 5a, which clearly shows the particle with spacings of 0.300 and 0.250 nm, and the crystal angle θ_1_ (0.300/0.250) of 90°, satisfying i‐C (110) and (111) planes. In addition, the black particle in Figure 5c has internal lattice stripes with a spacing of 0.210 nm close to the diamond (111) planes, that is a diamond particle ≈7 nm in size. Also, many graphite transiting to amorphous carbon is found in sample T15‐A500 (Figure S14, Supporting Information), especially in the edge regions, which is quite different from the intrinsic graphite, suggesting that Ta atoms contribute to the graphite to amorphous carbon transition. More importantly, there are some diamond grains found in this sample, meaning that graphite transits to diamond under as low a temperature of 500 °C.

With increasing the annealing temperature to 700 °C (sample T15‐A700), many amorphous carbon spheres are present in the sample (Figure 5d), which agrees with the SEM results (Figure S12b, Supporting Information). It is clear in Figure S13b (Supporting Information) that there are diffraction rings for different planes of graphite. Selecting the region e in Figure 5d for magnification, as shown in Figure 5e, we find that the particles with different contrasts, in which amorphous features are observed in the region of shallow contrast, and lattice stripes with a spacing of 0.300 nm, corresponding to the i‐C (110) planes, are present in the deeper contrast region. Moreover, the high magnification HRTEM image Figure 5f shows a black particle of 5 nm containing lattice stripes with a spacing of 0.210 nm, close to the diamond (111) planes. Thus, sample T15‐A700 forms many amorphous carbon spheres, some of which transit to i‐C. Meanwhile, diamond particles are found in graphite.

At the annealing temperature of 1000 °C, more spherical amorphous carbon particles are found in the sample T15‐A1000. At the same time, Figure S13c (Supporting Information) shows the diffraction of graphite and TaC, indicating more TaC starts to appear at 1000 °C. Enlarging the region h in Figure 5g, as shown in Figure 5h, it is obvious that there are some overlapping particles, including amorphous carbon spherical particles and black particles. In particular, there are lattice streaks with a spacing of 0.210 nm inside the black particle, corresponding to diamond (111) planes. The entire diamond particle is surrounded by graphite (002) planes. Similarly, a spherical particle with ≈6 nm in size is shown in Figure 5i, which has internal lattice spacing of 0.210 nm and a crystal angle of 70°, satisfying the cubic diamond (111) planes. It is also surrounded by a distorted graphite (002) lattice. Therefore, nanodiamond particles of 6–18 nm are present in sample T15‐A1000.

When the annealing temperature is increased further to 1200 °C (sample T15‐A1200), the nanoparticles in the sample decrease significantly (Figure 5j), which is consistent with the SEM results (Figure S12e, Supporting Information). It is clear that the diffraction information of the graphite and TaC are present in Figure S13d (Supporting Information). Enlarging the region k in Figure 5j, there are lattice stripes with a spacing of 0.197 nm and a crystal plane angle θ_1_ (0.190/0.190) of 80° inside the particle in Figure 5k. Meanwhile, the FT image has diffraction points corresponding to the crystal planes with spacings of 0.190, 0.150, and 0.130 nm. And the crystal angle θ_1_ (0.190/0.190) is 80°, θ_2_ (0.190/0.130) is 40°, and θ_3_ (0.190/0.150) is 50°. These planes satisfy the relationship to the hexagonal diamond (H‐D) crystal planes, corresponding to the H‐D (101), (102), and (110) planes, which proves that an H‐D particle with a size larger than 20 nm has been obtained. It is notable that a small amount of graphite (002) lattice as well as amorphous carbon regions remain at the edge of this particle. Also, in another region l of the sample, the high magnification HRTEM image Figure 5l displays a black particle of ≈12 nm in size, with internal lattice stripes with a spacing of 0.210 nm, close to the cubic diamond (111) planes, which is surrounded by residual graphite (002) crystal planes, consistent with the previous results. So, cubic and hexagonal diamonds surrounded by graphite (002) crystal planes are found in sample T15‐A1200.

These results reveal that few diamond phases are found in the samples at lower annealing temperatures, with particle sizes of 2–5 nm; as the annealing temperature increases, more diamond particles are found and the particle size increases to 6–20 nm, suggesting that the increase of annealing temperature is beneficial for the transformation from graphite into diamond. In addition, amorphous carbon spheres are found to form in the samples, and i‐C, as well as diamond structures, are formed inside them, suggesting that amorphous carbon can transform into i‐C and diamond under the action of Ta atoms.

### 2.4 Discussion of the Graphite/Diamond Phase Transition Mechanism

**Figure 6:**
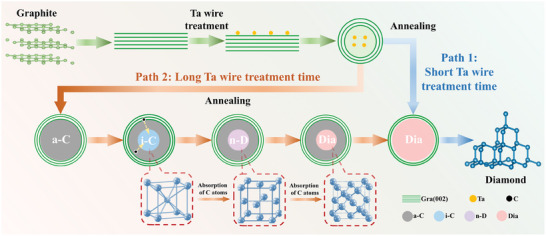
The phase transition process from graphite to diamond at ordinary pressure induced by Ta atoms.

The above results show that Ta atoms escaping from Ta wires are deposited on graphite substrates in the HFCVD system, forming graphite “circle” structures and amorphous carbon clusters; then, amorphous carbon spheres, i‐C, n‐D, and diamond are further formed after annealing with different processes. It confirms that monodispersed Ta atoms play a key role in the phase transition from graphite to diamond at ordinary pressure. Different concentrations of Ta are deposited on the graphite surface by different Ta wire treatment time, causing the process of graphite transition to diamond to be different under annealing. Specifically, at shorter Ta wire treatment time (5–10 min), the concentration of Ta is low, and monodisperse Ta atoms cause graphite to curl and form a separate “circle” structure, and the internal graphite is directly transformed into diamond (path 1 in **Figure** **6**). Also, the chemical bond formed between Ta atoms and carbon atoms is stronger than the van der Waals forces between graphite layers. This broke the local mechanical equilibrium.^[^ ^41^ ^]^ Moreover, the uneven distribution of Ta atoms leads to different stresses in various parts of graphite, increasing the surface energy, and changing the electronic structure and density distribution of carbon atoms.^[^ ^42^, ^43^ ^]^ All these changes lead to the formation of a “circle” structure in graphite to accommodate the new energy state. Within the “circle” structure, there are monodisperse Ta atoms, which decreases the phase transition potential barrier and transforms internal graphite into diamond directly. This observation is consistent with our previous theoretical calculations.^[^ ^28^, ^29^, ^44^ ^]^ Furthermore, the formed “circle” structure is similar to the carbon onion structure with a unique nanoscale pressure cell function, which creates a high‐pressure environment and stress concentration in the interior, causing the internal atoms to rearrange and form the diamond structure.^[^ ^45^, ^46^, ^47^ ^]^ Meanwhile, this structure has a layered structure similar to graphite, with sp^3^ defects on its surface reducing the barriers to diamond nucleation.^[^ ^46^, ^48^ ^]^ Also, the formation of the graphite “circle” structure causes an increase in the curvature of the carbon layers, contributing to the bonding transition.^[^ ^49^ ^]^

Extending the Ta wire treatment time (15–20 min), the concentration of Ta increases, and the number of monodisperse Ta atoms on the graphite surface increases. Under the effect of monodisperse Ta atoms and annealing, the graphite curls and forms amorphous carbon, which needs to be transformed into i‐C and n‐D first, and then finally into diamond (path 2 in Figure 6). The Ta atoms deposited by long‐term Ta wire treatment intensify the changes in interatomic forces, local stresses, surface energy and electronic structure of graphite.^[^ ^41^, ^42^ ^]^ Graphite experiences increased pulling forces from surrounding tantalum atoms of varying orientations, leading to the distortion of the graphite lattice in different directions. This results in the formation of stacked “circles” structures and even disrupts the original layered structure of graphite, reducing its orderliness and forming amorphous carbon. Amorphous carbon is known as unstable and is a nucleation site for diamonds.^[^ ^21^ ^]^ So, the formed amorphous carbon further transits to i‐C, n‐D, and diamond under the action of Ta atoms and annealing. In addition, it was reported that the presence of surrounding amorphous carbon and atomic hydrogen affected the surface remodeling of i‐C and n‐D, which played an important role in the stability of metastable diamond variant structures.^[^ ^50^, ^51^, ^52^, ^53^ ^]^ Subsequently, the i‐C and n‐D continue to refine their structure and grow by combining the surrounding carbon atoms from the amorphous carbon and graphite “circles”, finally transforming into a diamond. Due to the outermost layers of the graphite “circles” not transforming, diamond particles surrounded by graphite (002) planes are finally formed. It was also reported that at the initial stage of diamond preparation by HFCVD, amorphous carbon particles were first formed, and then gradually transformed into a stable i‐C structure.^[^ ^54^ ^]^ As the growth proceeded, a core–shell structure with n‐D as the core and i‐C as the shell was formed, and both had the same lattice orientation, suggesting that i‐C is able to transform into n‐D. Therefore, it indicates that amorphous carbon is an important intermediate process in the transition from graphite to diamond at ordinary pressure, and i‐C and n‐D are the important transition state structures for the transformation of amorphous carbon into diamond.

Based on the above results and analysis, we summarize the process of the atmospheric pressure phase transformation of graphite to diamond induced by monodisperse Ta atoms, as shown in Figure 6. This provides a novel strategy for understanding the mechanism of phase transformation from graphite to diamond induced by monodispersed Ta atoms at ordinary pressure.

## 3 Conclusion

In summary, we have achieved the transition of graphite to diamond at ordinary pressure by using monodispersed Ta atoms from metal Ta wires in the HFCVD system and obtained diamonds with the size of 5–20 nm. It is found that there are different processes in the graphite‐to‐diamond transition due to the concentration of Ta elements. At short Ta wire treatment time, the graphite surface forms a distinctive circle structure and directly transforms the internal graphite into a diamond. However, when the Ta wire treatment time is increased, the process becomes more complex: the graphite undergoes more obvious curling and transforms into amorphous carbon, then into the intermediate states i‐C, n‐D, and finally into diamond. Additionally, by extending the annealing time from 30 to 120 min, the i‐C (at 30 min) and n‐D (at 120 min) structures appear sequentially, as well as a large number of diamonds are obtained when annealing for 120 min. Meanwhile, annealing at a lower temperature of 500 °C leads to the formation of diamonds, as well as the number and size of diamonds significantly increase with annealing temperatures, reaching 20 nm at 1200 °C. So, this work provides a fresh idea for understanding the mechanism of graphite/diamond phase transition and new methods for preparing diamonds based on graphite or even other different carbon materials at ordinary pressure.

## 4 Experimental Section

### Experimental—Tantalum wire treatment

The surface of the pressed graphite flakes was successively smoothed with 2000 mesh and 4000 mesh sandpaper, and then polished with a polishing cloth. Then, it was soaked in ethanol solution and ultrasonically cleaned for 15 min, dried with nitrogen, and put into the HFCVD equipment. Hydrogen gas flow rate of 100 sccm, air pressure of 3.5 kPa, power of 400 W, and experimental time of 5, 10, 15, 20, and 25 min, to obtain Ta wire treatment samples at different time (named T5, T10, T15, T20, and T25).

### Experimental—Vacuum annealing

The samples with different Ta wire treatment time were sealed in a quartz tube and placed into the muffle furnace when the temperature reached 1100 °C and annealed for 30 min to obtain the annealed samples (named T5‐A1100, T10‐A1100, T15‐A1100, T20‐A1100, and T25‐A1100).

Kept the Ta wire treatment time at 15 min and changed the vacuum annealing time, annealed to 30, 60, 90, 120, and 150 min at 1100 °C, respectively, to obtain different annealing time samples (named T15‐A1100‐30, T15‐A1100‐60, T15‐A1100‐90, T15‐A1100‐120, and T15‐A1100‐150).

Kept the Ta wire treated to time at 15 min and changed the vacuum annealing temperature, annealed at 500, 700, 1000, and 1200 °C for 30 min, respectively, to obtain different annealing temperature samples (named T15‐A500, T15‐A700, T15‐A1000, and T15‐A1200).

The experimental conditions and sample names for each section are summarized in Table S2 (Supporting Information).

### Characterizations

FEI's Field Emission Scanning Electron Microscope (FESEM, model Nova Nano 450) was used to observe the morphology and Energy‐dispersive X‐ray spectroscopy (EDS) of the sample surface; FEI's High‐Resolution Transmission Electron Microscope (HRTEM, model TalosS‐FEG) was used to observe the microstructure and EDS of the sample surface; FEI's spherical aberration‐corrected transmission electron microscope (ACTEM, model Thermo Scientific Spectra 300) was used to obtain microstructural information, electron energy loss spectroscopy (EELS) data and high‐angle annular dark field (HAADF) maps of the sample surface.

## Conflict of Interest

The authors declare no conflict of interest.

## Supporting information



Supporting Information

## Data Availability

The data that support the findings of this study are available from the corresponding author upon reasonable request.
