# Supplementary material for: Phase Transition Process of Graphite to Diamond Induced by Monodispersed Tantalum Atoms at Ordinary Pressure
Source: Adv Sci (Weinh). 2025 Jan 22;12(10):2411504. doi: 10.1002/advs.202411504 (PMC11905102; doi:10.1002/advs.202411504)
Supplement: Supplementary file 1 — Supporting Information [file ADVS-12-2411504-s001.docx]

**Supplementary Information**

**Phase Transition Process of Graphite to Diamond Induced by Tantalum Atoms at Ordinary Pressure**

Zhiguang Zhu^1, 2, 3, 4^, Chengke Chen^1, 2, 3, 4^, Shaohua Lu^1, 2, 3, 4^,

Xiao Li^1, 2, 3, 4^, Xiaojun Hu^1, 2, 3, 4, *^

^1^ College of Materials Science and Engineering, Zhejiang University of Technology, Hangzhou, 310014, P. R. China

^2^ Moganshan Diamond Research Center, De Qing, Hu Zhou, 313200, P. R. China

^3^ Diamond Joint Research Center for Zhejiang University of Technology and Tanghe Scientific & Technology Company, De Qing, Hu Zhou, 313200, P. R. China

^4^ Moganshan Institute ZJUT, De Qing, Hu Zhou, 313200, P. R. China

*Correspondence and requests for materials should be addressed to [huxj@zjut.edu.cn](mailto:huxj@zjut.edu.cn)


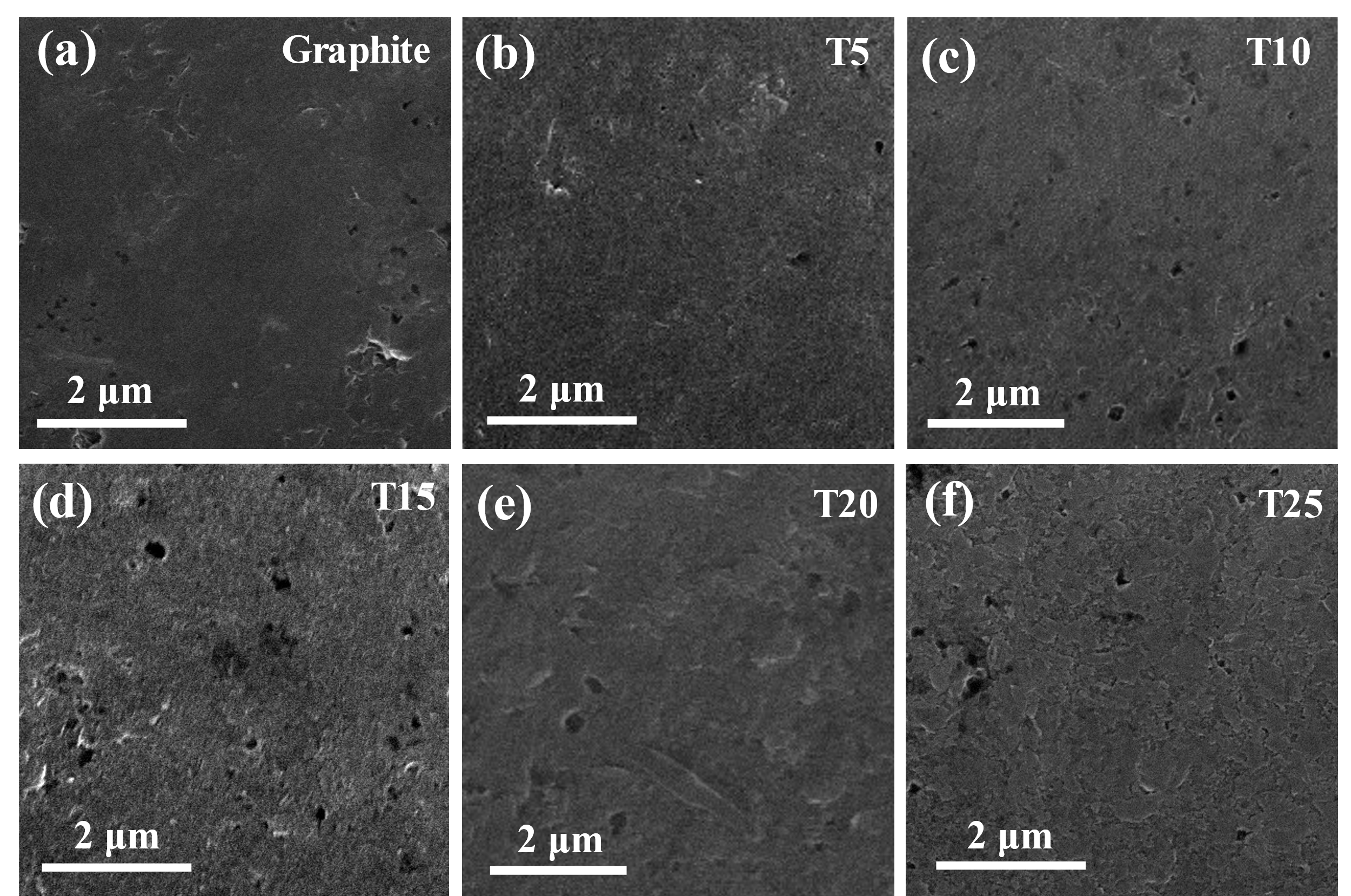


Figure S1 SEM images of samples with different Ta wire treatment time: (a) intrinsic graphite; (b) 5 min; (c) 10 min; (d) 15 min; (e) 20 min; (f) 25 min.


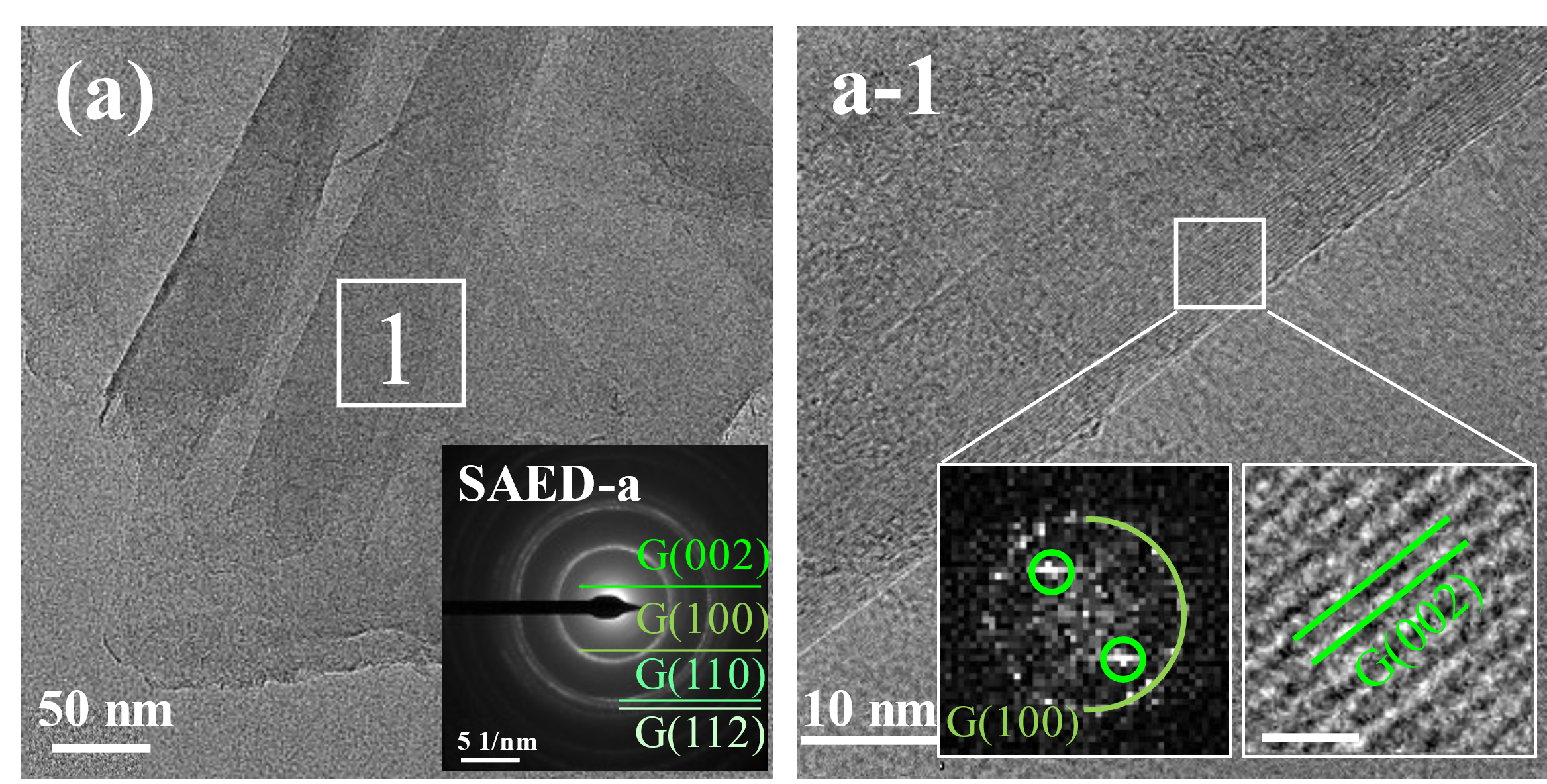


Figure S2 (a) TEM images of the intrinsic graphite and (a-1) corresponding magnified image and FT image. The white line in the figure indicates the 1 nm scale.


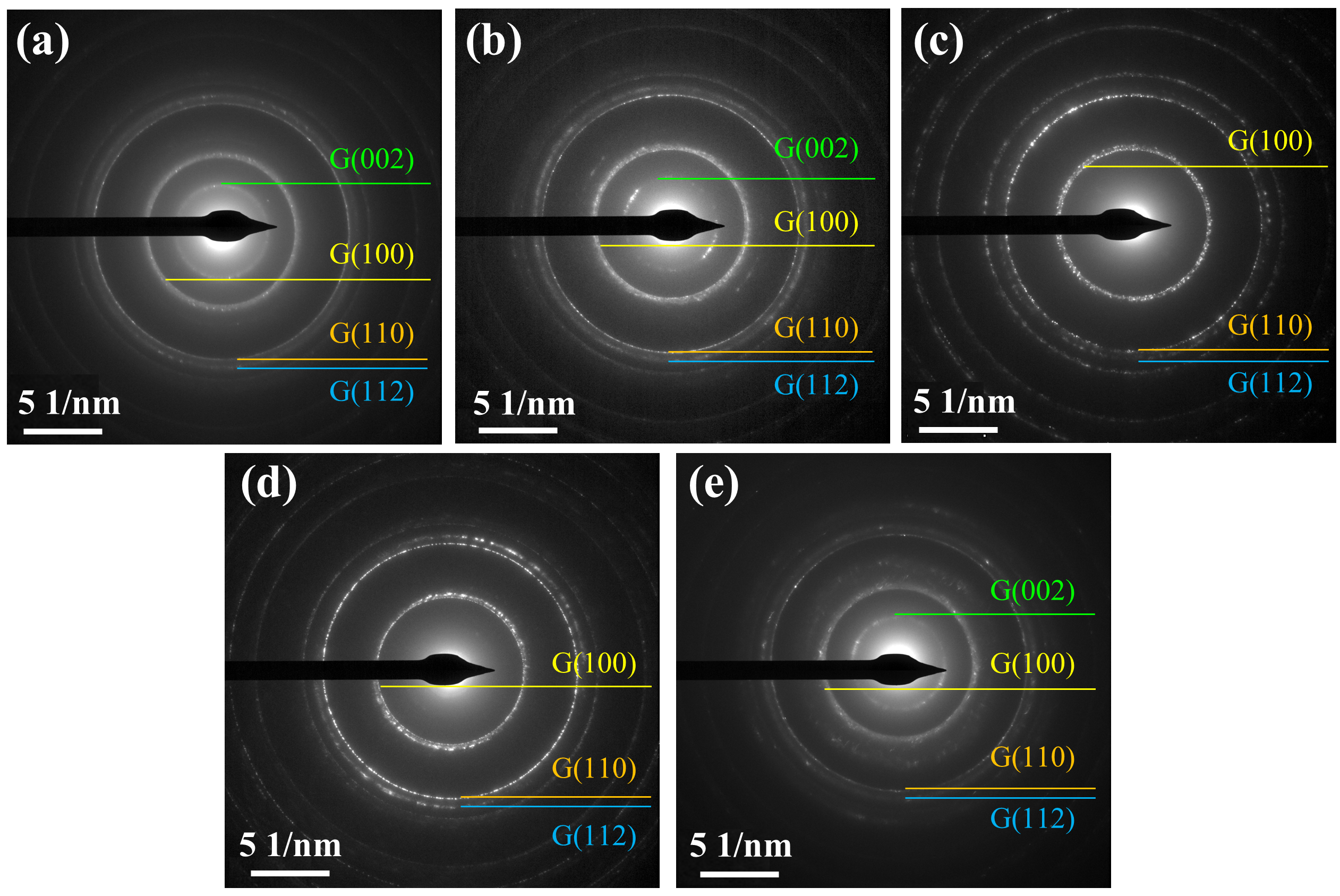


Figure S3 Selected area electron diffraction (SAED) images of samples with different Ta wire treatment time: (a) 5 min; (b) 10 min; (c) 15 min; (d) 20 min; (e) 25 min.


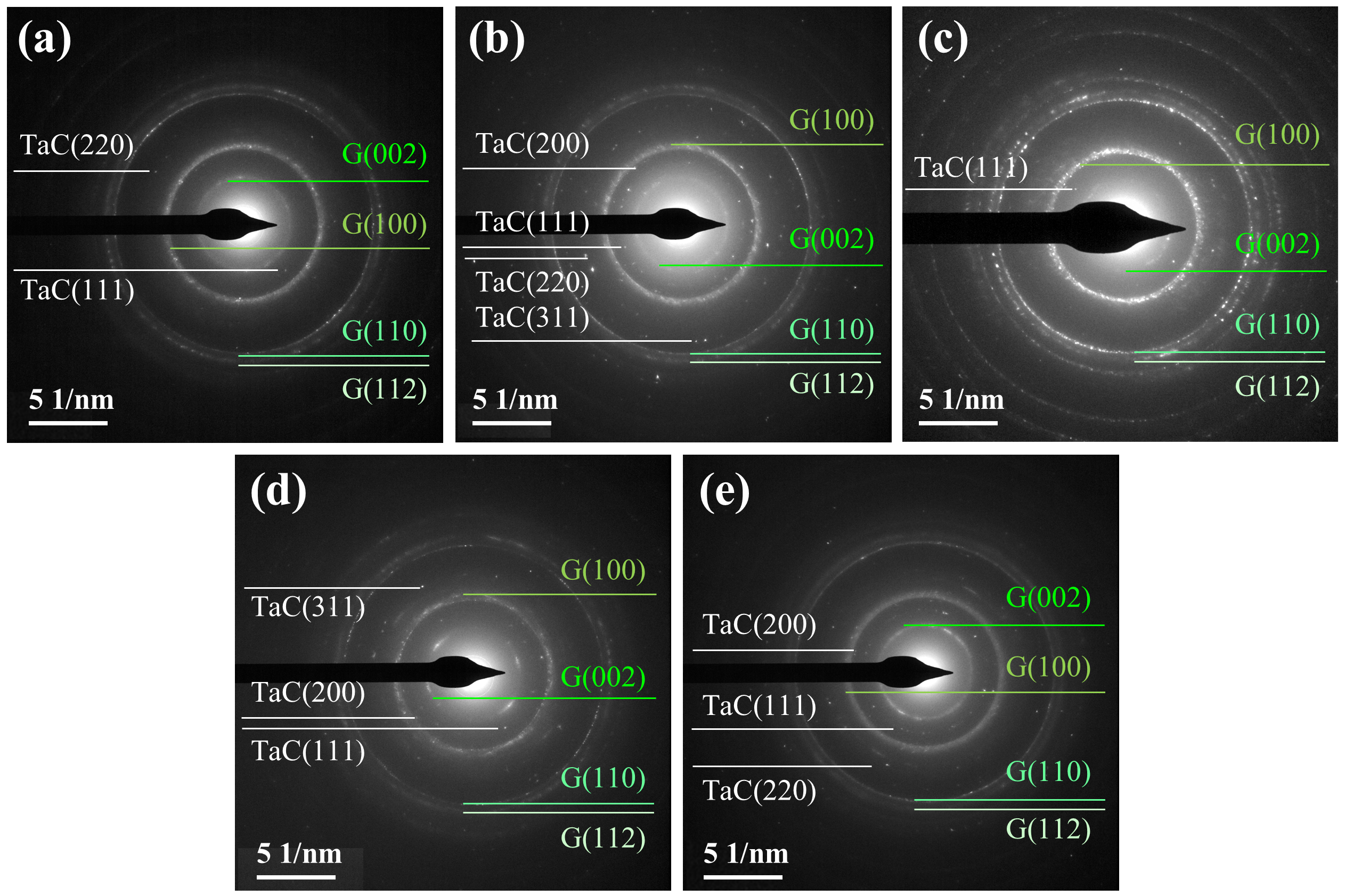


Figure S4 Selected area electron diffraction (SAED) images of samples with different Ta wire treatment times after annealing at 1100 °C for 30 minutes: (a) 5 min; (b) 10 min; (c) 15 min; (d) 20 min; (e) 25 min.


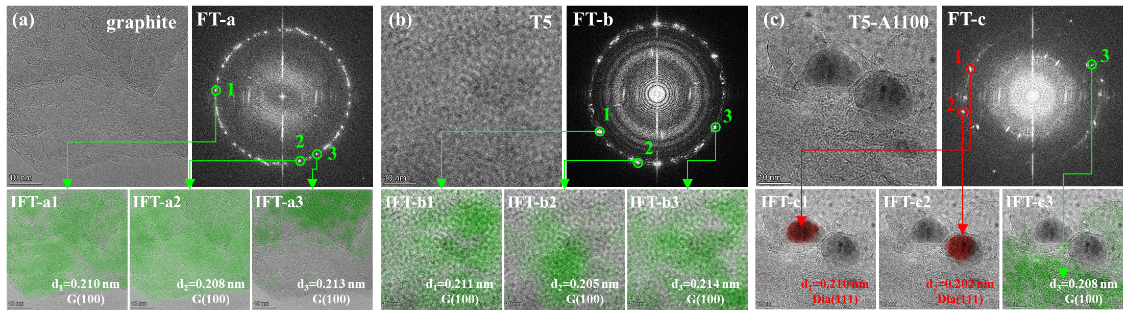


Figure S5 HRTEM, Fourier Transform and Inverse Fourier Transform images of different samples: (a) intrinsic graphite, (b) sample treated with tantalum filament for 5 minutes, named T5, (c) sample treated with tantalum filament for 5 minutes and then annealed, named T5-A1100.


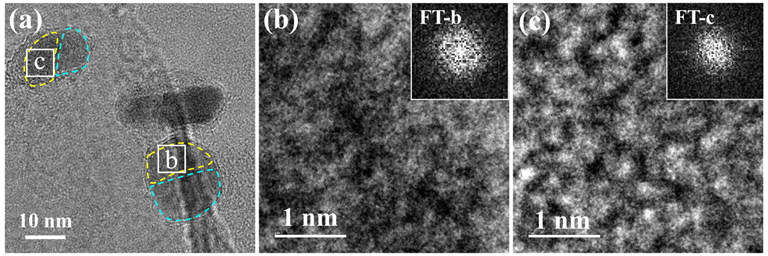


Figure S6 HRTEM image of the amorphous carbon region inside the particles formed in sample T15-A1100.


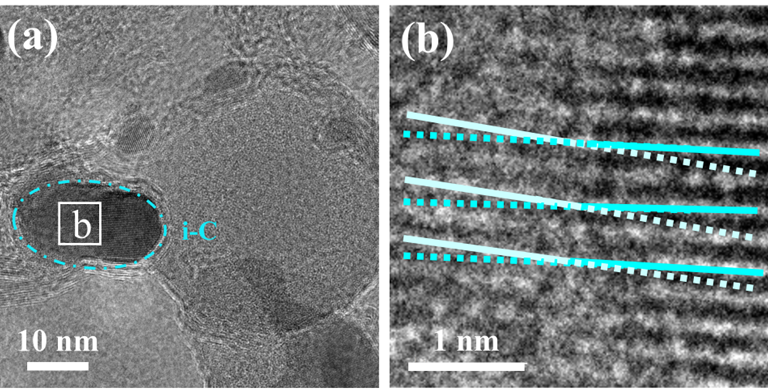


Figure S7 HRTEM image of the transition region inside the i-Carbon particle formed in sample T20-A1100.


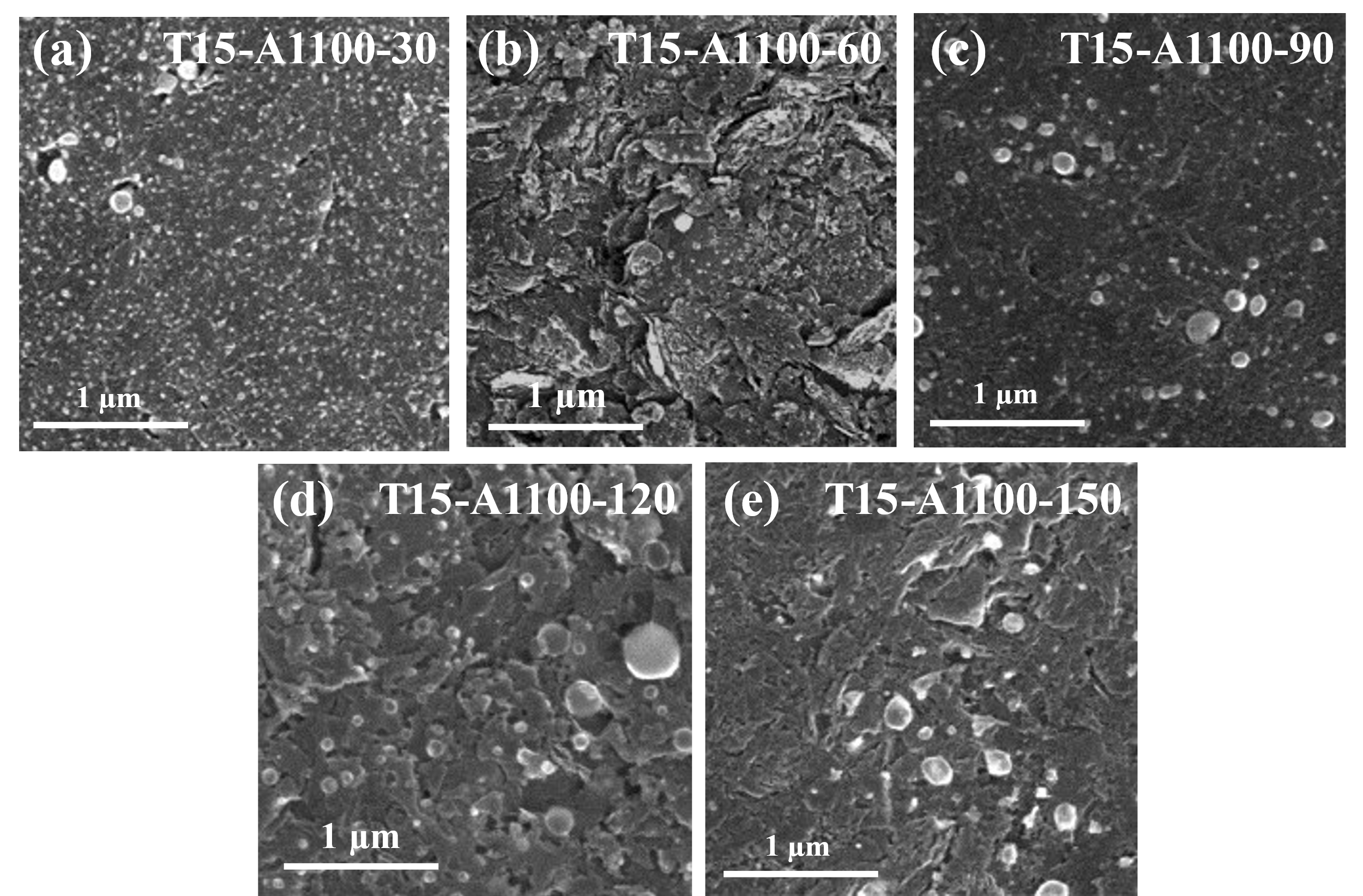


Figure S8 SEM images of samples with different annealing time: (a) 30 min; (b) 60 min; (c) 90 min; (d) 120 min; (e) 150 min.

Table S1 Comparison of experimentally observed spacing values for different crystal planes of cubic diamond, i-C and n-D


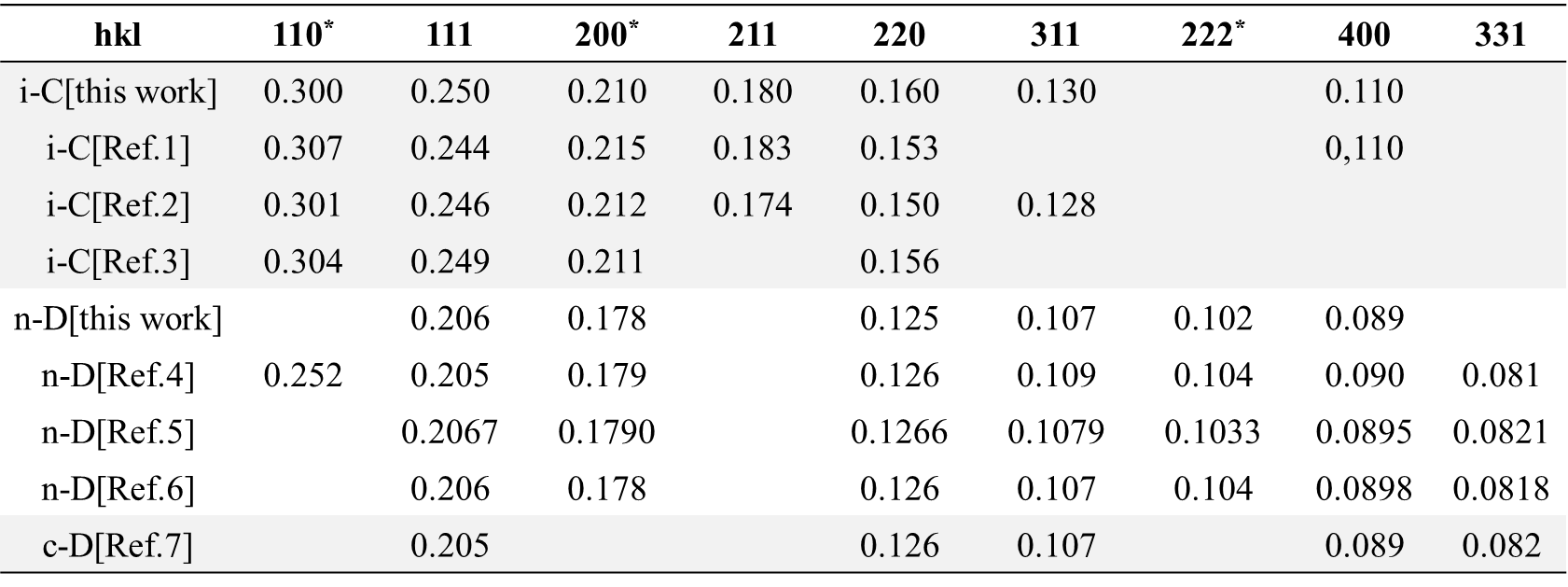


The superscript “*” stands for forbidden diffraction of cubic diamond. The unit of interplanar spacing is nm.


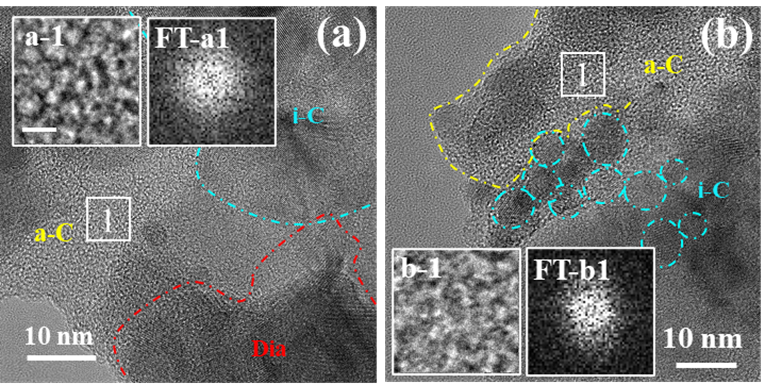


Figure S9 HRTEM image of the amorphous carbon region formed in sample T15‑A1100-30.


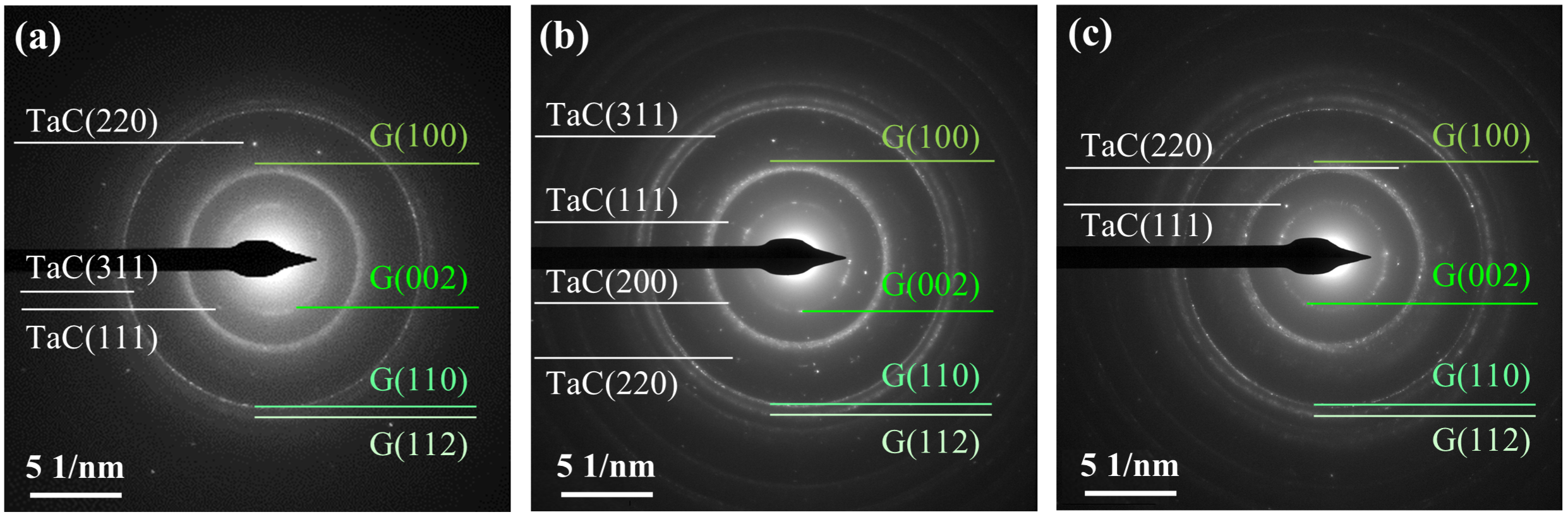


Figure S10 Selected area electron diffraction (SAED) images of samples with different annealing time: (a) 60 min; (b) 90 min; (c) 120 min.


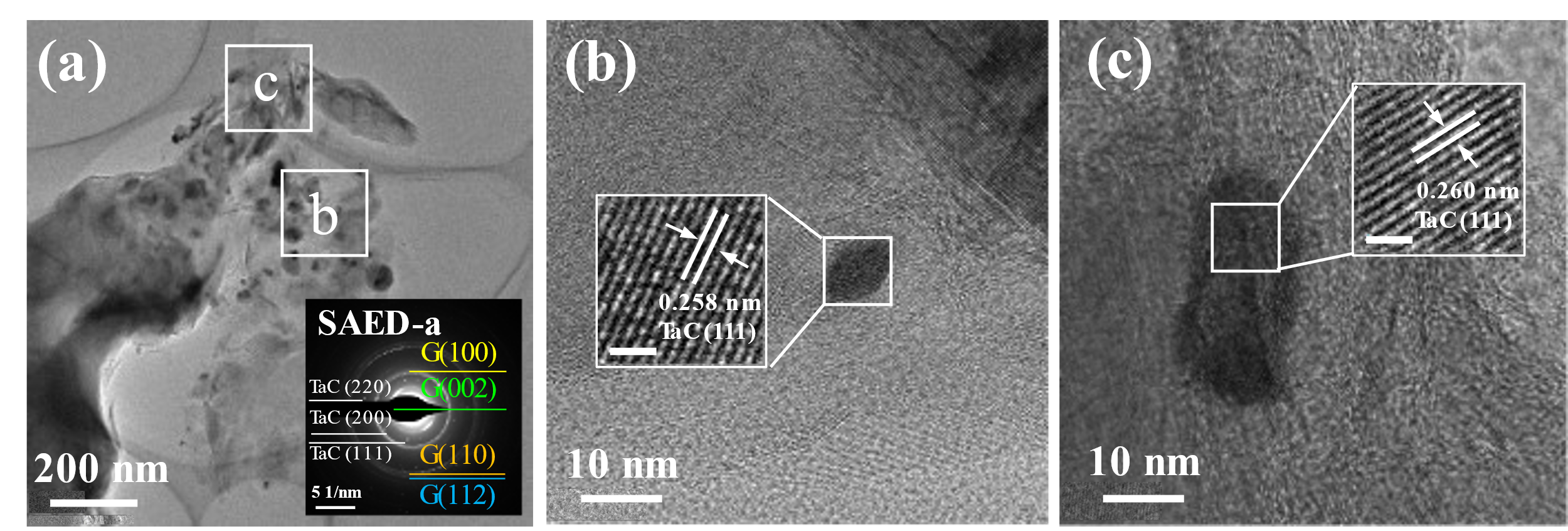


Figure S11 (a) TEM image of annealed 150 min sample and (b, c) corresponding magnified image. The white line in the figure indicates the 1 nm scale.


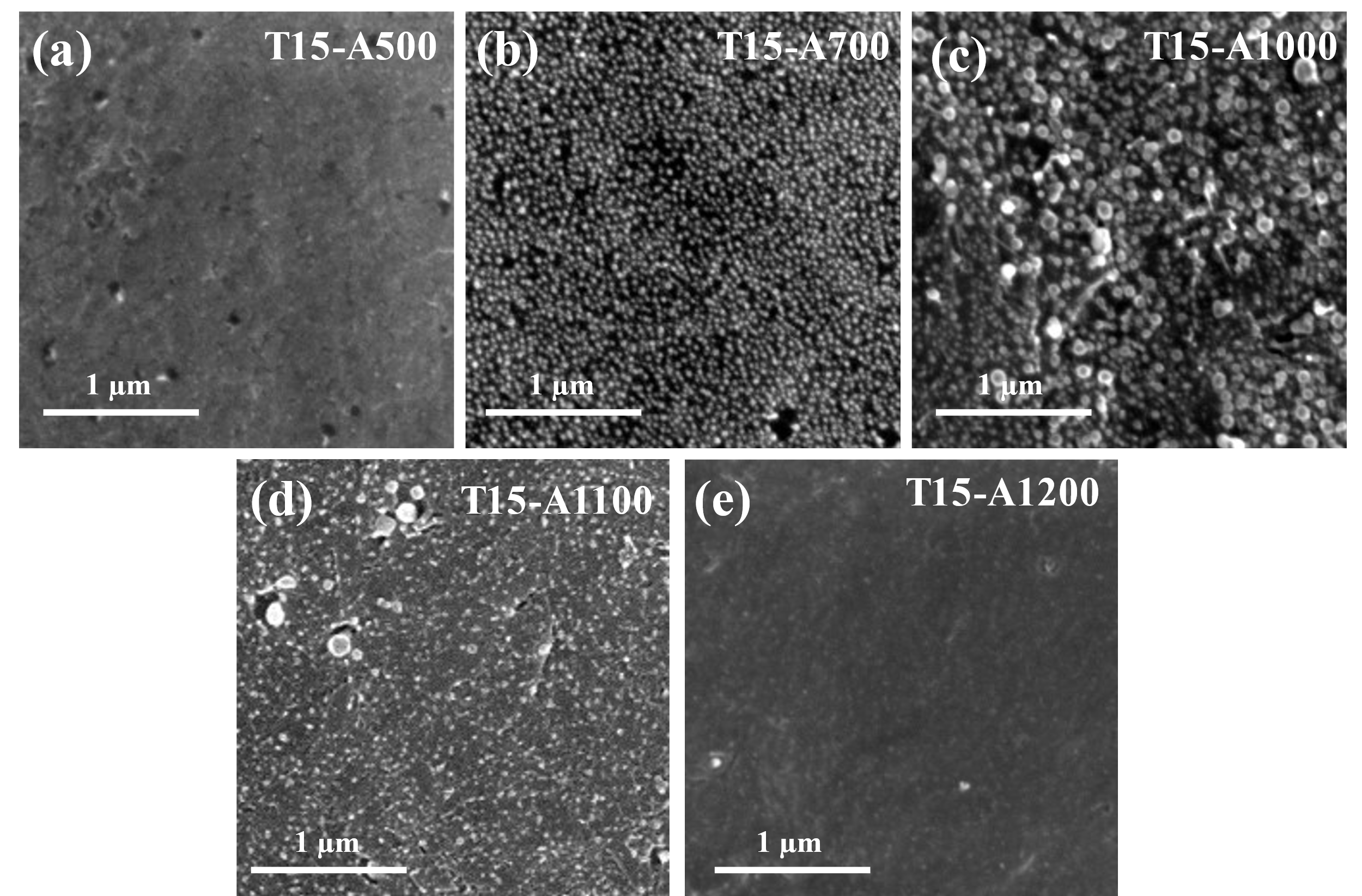


Figure S12 SEM images of samples with different annealing temperatures: (a) 500 ℃; (b) 700 ℃; (c) 1000 ℃; (d) 1100 ℃; (e) 1200 ℃.


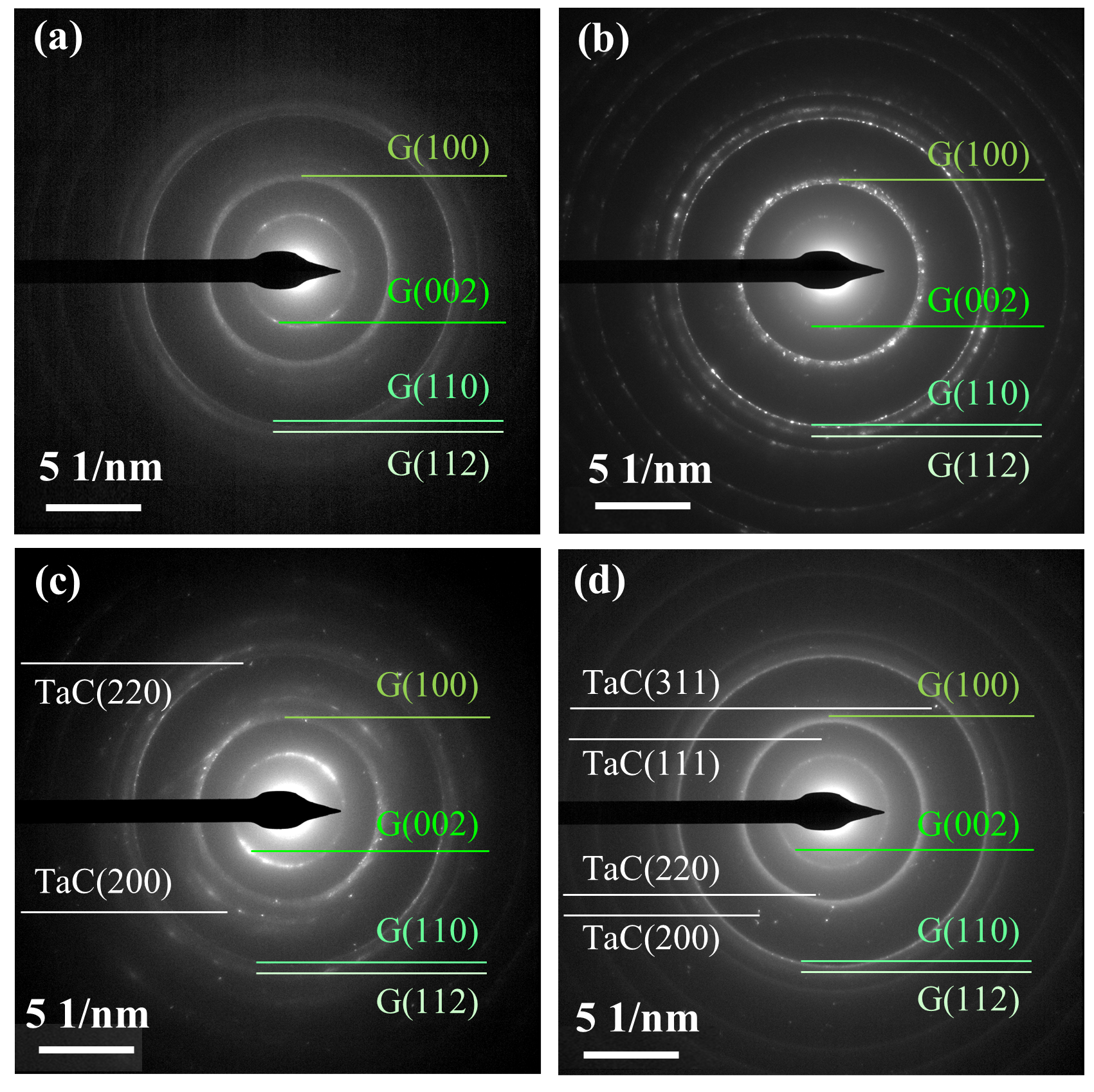


Figure S13 Selected area electron diffraction (SAED) images of samples with different annealing temperatures: (a) 500 ℃; (b) 700 ℃; (c) 1000 ℃; (d) 1200 ℃.


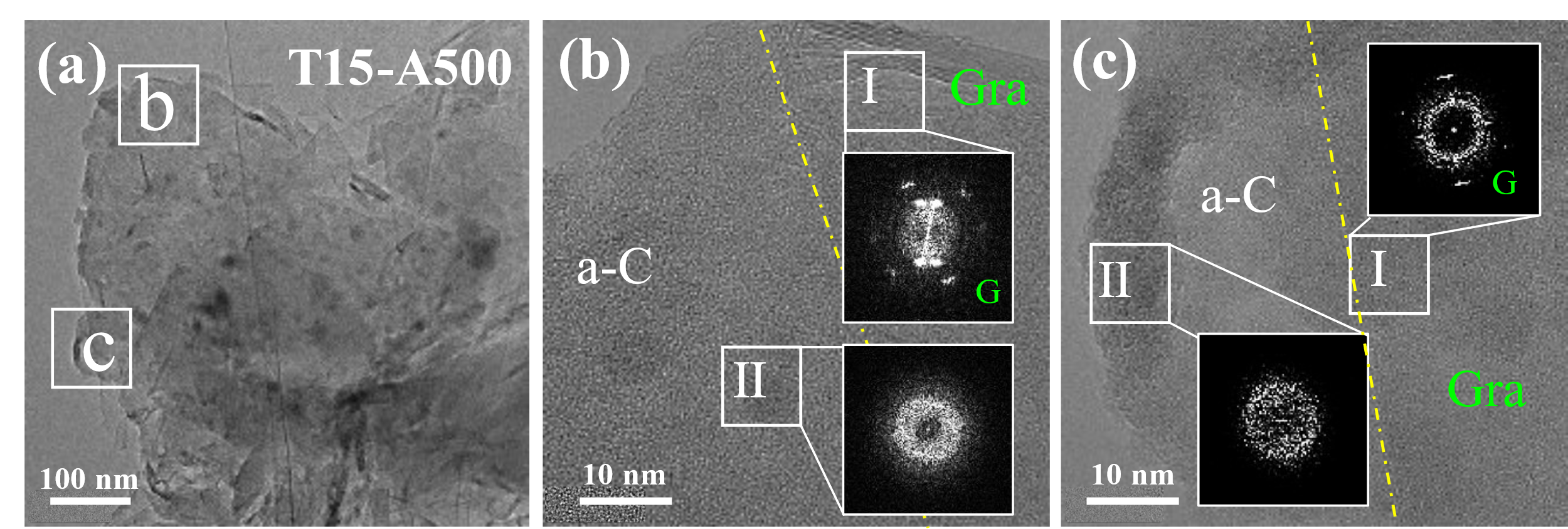


Figure S14 (a-c) TEM images of the sample T15-A500 and corresponding FT images.

Table S2 Summary of the samples obtained from different treatment conditions


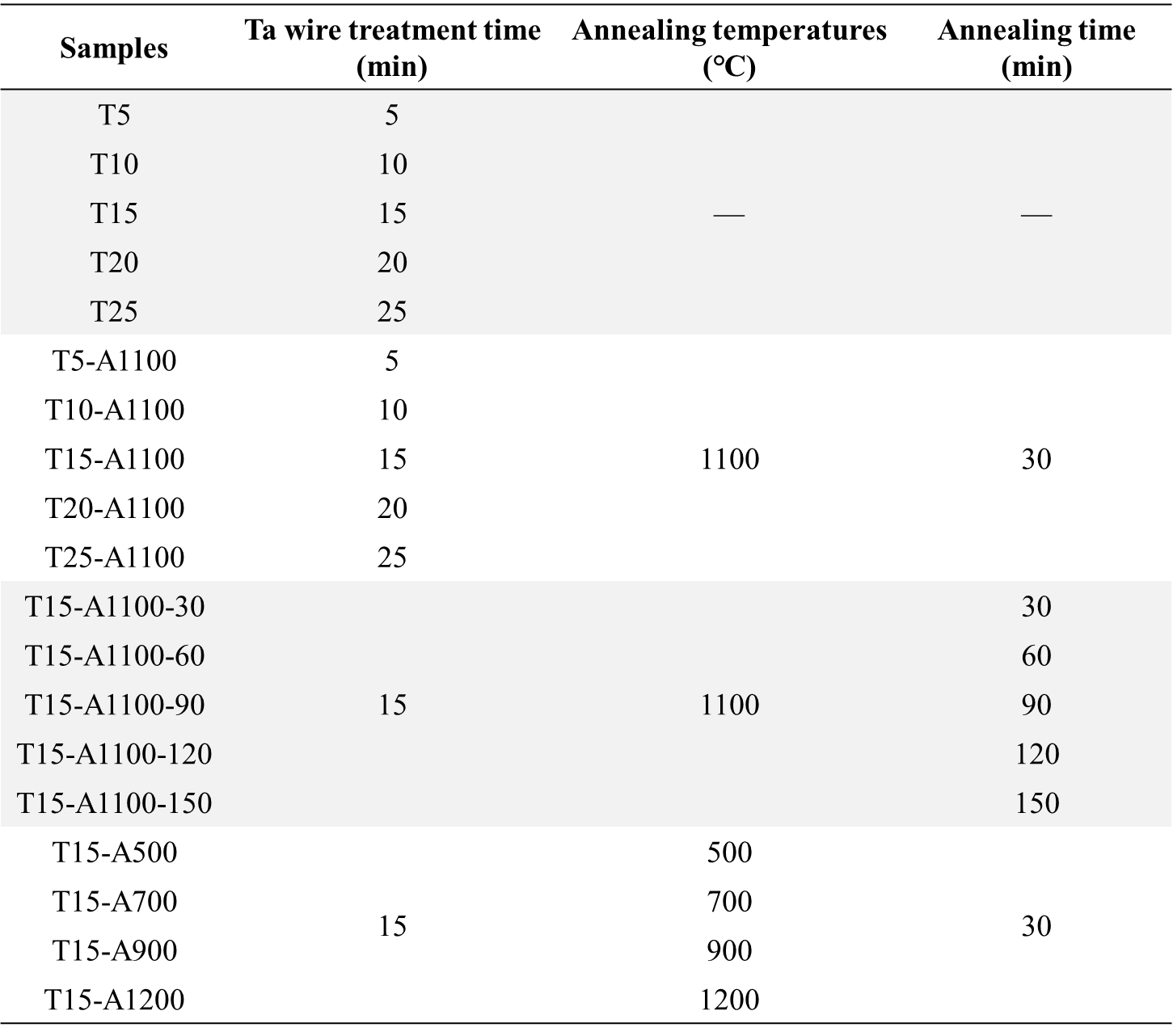


**References**

[1] I. Konyashin, D. J. Frost, D. Sidorenko, A. Orekhov, E. A. Obraztsova, T. A. Sviridova, Diam. Relat. Mater. 2020, 109, 108017.

[2] H. Vora, T. J. Moravec, Journal of Applied Physics 1981, 52, 6151.

[3] C. G. Park, J. W. Yang, N. M. Hwang, Electron. Mater. Lett. 2023, 19, 316.

[4] D. J. Dai, Y. Y. Li, J. Y. Fan, Carbon 2021, 179, 133.

[5] X. X. Ma, X. Y. Liu, Y. Y. Li, X. N. Xi, Q. Q. Yao, J. Y. Fan, Nanotechnology 2020, 31, 505712, 505712.

[6] H. Hirai, K. Kondo, Science 1991, 253, 772.

[7] A. W. Hull, Physical Review 1917, 10, 661.
